# Microbial conversion of ethanol to high-value products: progress and challenges

**DOI:** 10.1186/s13068-024-02546-w

**Published:** 2024-08-19

**Authors:** Manman Sun, Alex Xiong Gao, Xiuxia Liu, Zhonghu Bai, Peng Wang, Rodrigo Ledesma-Amaro

**Affiliations:** 1 Key Laboratory of High Magnetic Field and Ion Beam Physical Biology, Hefei Institutes of Physical Science, Chinese Academy of Sciences, Hefei, 230031 China; 2 Institute of Hefei Artificial Intelligence Breeding Accelerator, Hefei, 230000 China; 3 Department of Bioengineering and Imperial College Centre for Synthetic Biology, Imperial College London, London, SW7 2AZ UK; 4 Division of Life Science, The Hong Kong University of Science and Technology, Hong Kong, 999077 China; 5 National Engineering Research Center of Cereal Fermentation and Food Biomanufacturing, Jiangnan University, Wuxi, 214112 China

**Keywords:** Renewable carbon source, Ethanol, Biomanufacturing, Sustainability, Acetyl-CoA, Ethanol metabolism, Stress response

## Abstract

Industrial biotechnology heavily relies on the microbial conversion of carbohydrate substrates derived from sugar- or starch-rich crops. This dependency poses significant challenges in the face of a rising population and food scarcity. Consequently, exploring renewable, non-competing carbon sources for sustainable bioprocessing becomes increasingly important. Ethanol, a key C2 feedstock, presents a promising alternative, especially for producing acetyl-CoA derivatives. In this review, we offer an in-depth analysis of ethanol's potential as an alternative carbon source, summarizing its distinctive characteristics when utilized by microbes, microbial ethanol metabolism pathway, and microbial responses and tolerance mechanisms to ethanol stress. We provide an update on recent progress in ethanol-based biomanufacturing and ethanol biosynthesis, discuss current challenges, and outline potential research directions to guide future advancements in this field. The insights presented here could serve as valuable theoretical support for researchers and industry professionals seeking to harness ethanol's potential for the production of high-value products.

**Graphic Abstract:**

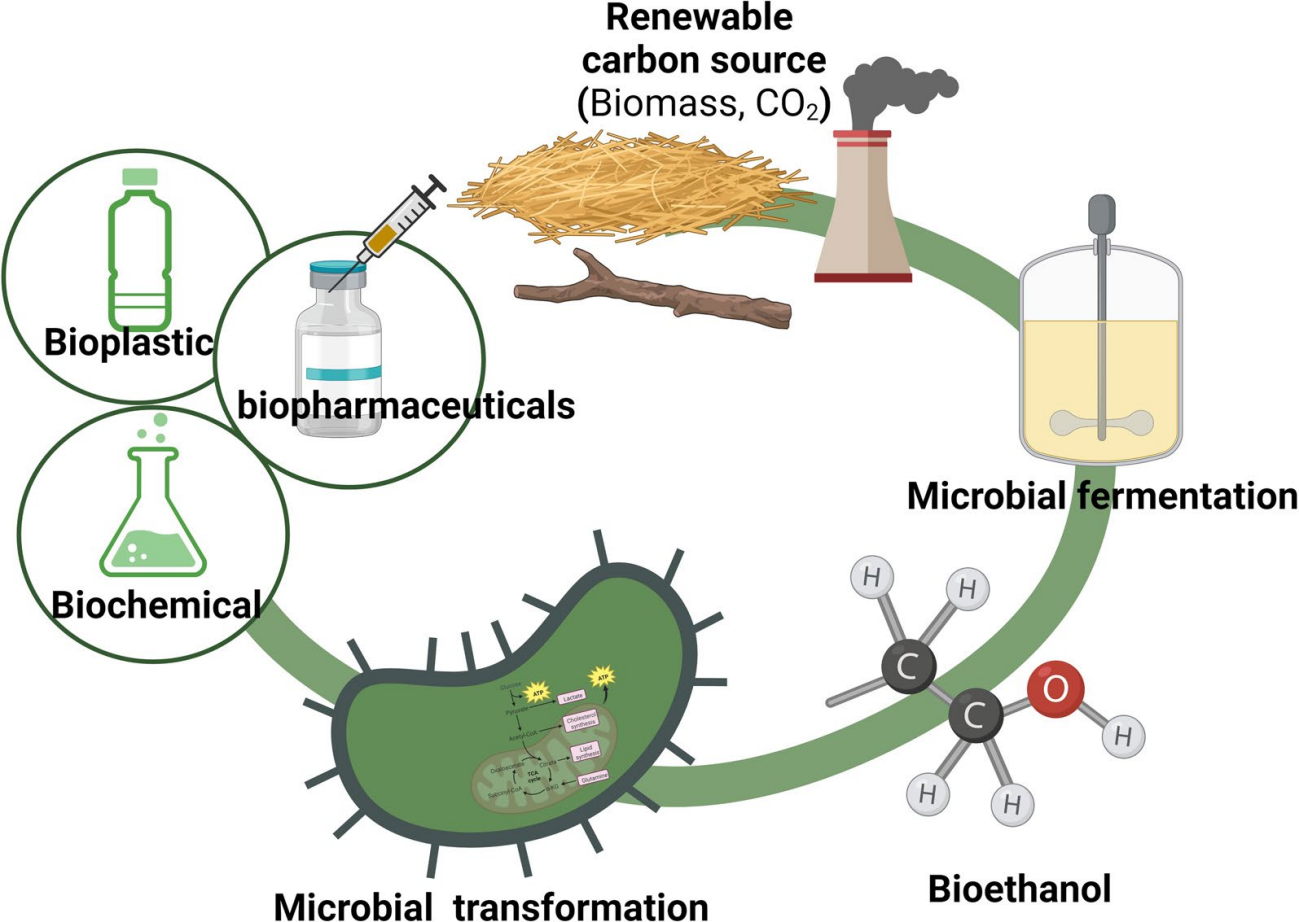

## Highlight

- Highlights the characteristics of ethanol as a carbon source in bioprocessing.
- Summarizes microbial ethanol metabolism, stress responses, and tolerance mechanisms.
- Reviews recent progress in ethanol-based biomanufacturing and ethanol biosynthesis.

## Introduction

Carbon, the essential building block of life, plays a pivotal role in the growth and reproduction of all organisms, including microbes. Therefore, the selection of carbon sources becomes paramount in applied fields such as bioprocessing and biomanufacturing, where the choice of carbon source significantly impacts both process efficiency and product synthesis [1, 2]. For industrial fermentation, the ideal carbon sources should exhibit high purity, ensure plentiful availability for a steady supply, and be compatible with the specific requirements of the microorganisms used [3]. This has stimulated the exploration of a diverse range of carbon sources in industrial biotechnology, spanning from carbohydrates, triglycerides, and derivatives, to alcohols, and even hydrocarbons [3].

Carbohydrates such as glucose and sucrose are the predominant substrates utilized in industrial biotechnology [4]. As these carbohydrates often derived from starch-containing crops, such dependency has raised concerns related to food scarcity and environmental crises [5]. Therefore, the exploration of alternative, renewable, and non-food carbon sources has become increasingly important. Significant progress has been made in the development of various non-food renewable feedstocks, including lignocellulosic biomass, plastics, and industrial waste (e.g., molasses, glycerol, and food waste) [6–8]. In recent years, simple carbon compounds, especially C1 (e.g., CO, CO_2_, methane, methanol, and formate) and C2 substrates (e.g., ethanol and acetate) have drawn great attention due to their natural abundance, low production cost, and easy availability as an industrial waste and byproduct [7, 9].

While exciting progress has been made in isolating methylotrophs and engineering nonnative microbes for the efficient utilization of C1 substrates in the production of high-value chemicals such as alcohols, organic acids, and amino acids, the limited availability of genetic tools and the complexity of assimilation pathways continue to present significant challenges [9–11]. In contrast, the assimilation pathway of C2 molecules into the central metabolism is notably simpler and more efficient [12]. Acetate and ethanol, two primary feedstocks in C2-biomanufacturing, have shown great promise [12, 13]. Ethanol has recently gained attention as a sustainable carbon source due to its unique advantages in producing acetyl-CoA-derived compounds [13, 14]. As a result, significant progress has been made in sustainable ethanol production and its biotechnological conversion into value-added products.

This review aims to highlight the potential of ethanol as a renewable feedstock for future industrial biotechnology. We provide a comprehensive overview of the characteristics and metabolism of ethanol as a carbon source, summarize microbial responses and tolerance mechanisms to ethanol stress, and review the advancement in ethanol-based biomanufacturing and bioethanol synthesis. This will provide a valuable solution and reference point for achieving efficient and sustainable bioprocessing using ethanol as a feedstock.

## The characteristics of ethanol as a carbon source in biomanufacturing

### The intrinsic properties of ethanol

Ethanol (PubChem CID: 702), also known as ethyl alcohol or simply alcohol, is an organic compound with the molecular formula C_2_H_5_OH. It consists of a two-carbon chain with a single hydroxyl (–OH) group attached to one carbon atom, while the rest of the bonds are occupied by hydrogen atoms. Ethanol is a clear, colorless, and volatile liquid at room temperature and pressure. Due to its smaller molecular size and weaker intermolecular forces, it has a lower boiling point (78 °C) than water. This characteristic facilitates its separation from other substances through distillation, particularly those with significantly different boiling points. Ethanol possesses high solubility in water, as the hydrogen bonds formed between ethanol and water molecules allow them to mix readily in different concentrations. Furthermore, the hydroxyl group in ethanol enables it to engage in lots of chemical reactions, such as esterification and dehydration.

### Advantages of ethanol as a carbon source

The association between ethanol and microorganisms has a long-standing history in human activities, tracing back to when our ancestors inadvertently fermented fruits to produce an intoxicating brew. This relationship has since evolved, with ethanol being used as a sole or co-carbon source in various microbial fermentation processes, such as the production of vinegar [13].

**Fig. 1:**
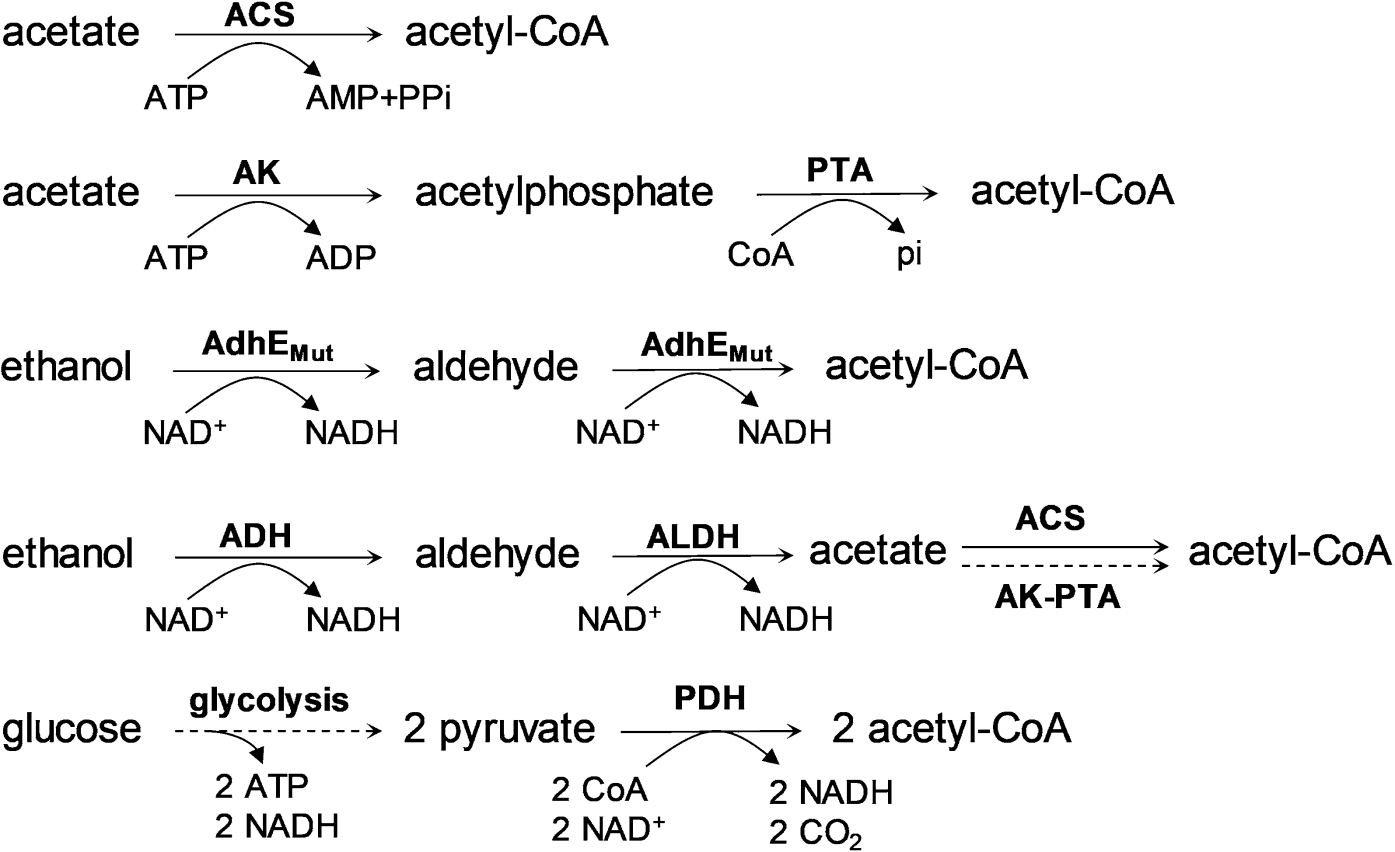
Metabolic pathway for the convention of acetate, ethanol, and glucose to acetyl-CoA. Single steps are represented by solid lines, while multiple steps are represented by dotted lines. The involved enzymes are as follows: ACS, AMP-forming acetyl-CoA synthetase; AK, acetate kinase; PTA, phosphotransacetylase; AdhE_Mut_, bifunctional acetyl-CoA reductase with two amino acid mutations (A267T/E568K); ADH, alcohol dehydrogenase; ALDH, aldehyde dehydrogenase; PDH, pyruvate dehydrogenase

Ethanol is a more environmentally sustainable carbon source than glucose when it is derived from raw materials such as agricultural and industrial waste or forest residues [8]. The emergence of fourth-generation bioethanol could further amplify this potential. This advanced form of bioethanol enables the direct synthesis of ethanol from CO_2_, aligning with global sustainability goals by providing a dual benefit of energy production and environmental remediation [15–17]. The metabolic pathway of ethanol is also simpler than that of glucose, which results in fewer by-products during processes like fermentation using genetically modified *Escherichia coli*, thereby simplifying downstream processing [14]. Moreover, ethanol exhibits superior energy density compared to glucose. The complete oxidation of 1 g ethanol to CO_2_ and H_2_O in bacteria generates 0.326 mol ATP, while 1 g glucose can only produce 0.178 mol ATP [14] (Table 1). The catabolism of ethanol to acetyl-CoA also exhibits superior atomic economy, with no carbon loss, resulting in 100% carbon recovery [14] (Fig. 1).

**Table 1:** Comparison of several carbon sources

	Ethanol	Glucose	Acetate	Methanol	Methane	Carbon dioxide
Formula	C_2_H_5_OH	C_6_H_12_O_6_	C_2_H_5_COOH	CH_3_OH	CH_4_	CO_2_
Molecular weight	46	180	60	32	16	44
Carbon content (%)	52.1	40	40.7	37.5	74.9	27.3
State	liquid	liquid	liquid	liquid	gas	gas
Water solubility (g/L)^a^	infinite	909	1,233	infinite	0.023	1.69
BOD^b^	166	56	–	88	–	–
FHG^c^	16000	4900	–	9700	–	–
ATP (mol/g)	0.326	0.178	0.133	–	–	–
NADH^d^	2	4	0	–	–	–
Carbon recovery (%)^e^	100	66.67	100	–	–	–
Price($/ton)^a^	250–350	300–400	300–450	150–300	200–320	0–80

^a^[6]

^b^ Biochemical oxygen demand (g/m^3^·h)

^c^ Fermentation heat generation (kcal/m^3^·h)

^d^ NADH generated from the conversion of one molecule substrate to acetyl-CoA

^e^ Carbon recovery rate during the catabolic process

In a comparison with C2 substrate, i.e., acetate, ethanol is non-corrosive and has a higher energy density (0.133 mol ATP/g acetate) [14] (Table 1). Ethanol is a more practical and easily controllable option for various bioprocessing applications due to its minimal impact on the pH of the fermentation broth, reducing the need for intensive pH control measures [7]. In addition, the conversion of ethanol to acetyl-CoA generates two additional NADH molecules compared to acetate. The AdhE^A267T/E568K^ mutant metabolic pathway even produces two more NADH without requiring an additional energy supply [14] (Fig. 1).

Ethanol also presents significant advantages when compared with C1 substrates. The metabolic assimilation of ethanol is relatively simple, and many microorganisms naturally possess ethanol metabolic pathways, simplifying the metabolic engineering process [9]. Unlike gaseous C1 sources (e.g., CO, CO_2_, and CH_4_), ethanol is a stable liquid at room temperature. This greatly facilitates its handling, storage, and transport. Furthermore, compared to the most widely used C1 carbon source methanol, ethanol is less toxic to microorganisms [18]. As a normal component of the human diet, ethanol also provides an extra margin of safety when used as a carbon source.

### Characteristics of bioprocesses using ethanol as a carbon source

The bioprocess using ethanol as a carbon source has several characteristics. For instance, the use of ethanol is generally associated with a slower growth rate and reduced biomass yield as compared to glucose-based processes [1, 19]. Moreover, high ethanol concentrations can induce ethanol stress and cause substrate inhibition to the microorganisms, necessitating a careful balance in feed strategies to prevent microbial stress while promoting efficient growth. Ethanol's higher carbon and lower oxygen content translates into an increased oxygen demand during ethanol-based bioprocess, requiring adjustments to the aeration strategy [1]. Furthermore, due to ethanol's higher calorific value, its metabolism produces more heat, potentially demanding additional cooling measures to maintain an optimal process temperature.

## Microbial ethanol metabolic pathways

The widely used carbon source in industrial biotechnology can be broadly categorized into sugars, organic acids, and alcohols. Microorganisms metabolize these carbon sources via different metabolic pathways. For instance, simple sugars like glucose and fructose are metabolized through the glycolytic pathway, which produces pyruvate that subsequently enters the tricarboxylic acid (TCA) cycle [14]. Organic acids, such as acetate and pyruvate, are directly converted by specific enzyme systems to acetyl-CoA, which then enter the TCA and glyoxylate cycles [7]. Alcohol metabolism typically involves oxidation–reduction reactions, where specific dehydrogenases oxidize alcohols to their corresponding aldehydes or ketones. These intermediates are then oxidized to acids, which then convert to acyl-CoA and funneled into the TCA and glyoxylate cycles [20]. Among various alcohols, ethanol metabolism serves as an example of this general pathway [1, 21].

Ethanol is primarily metabolized into acetate through the alcohol dehydrogenase (ADH) pathway. This pathway encompasses two key reactions: ethanol is first converted into acetaldehyde by ADH and then further transformed into acetate by aldehyde dehydrogenase (ALDH). Different organisms rely on distinct coenzymes and enzymatic systems for this process. For example, *Corynebacterium glutamicum*, *Aspergillus nidulans*, and yeasts use an NAD^+^-dependent ADH and a NAD^+^/NADP^+^-dependent ALDH. During this process, two molecules of reducing force (2 NADH or 1 NADH and 1 NADPH) can be generated [1, 22, 23]. While Pseudomonads and acetic acid bacteria use a pyrroloquinoline quinone (PQQ)-dependent ADH and a NAD^+^/PQQ-dependent ALDH [1]. In the ADH pathway, ADH is the most well-studied enzyme. Research has demonstrated that the formation of ADH's active site necessitates the involvement of certain metals. For instance, in several fungi, including *S. cerevisiae*, *Scheffersomyces stipites*, and *Kluyveromyces lactis*, the formation of the active site of ADH requires the presence of Zn^2+^ [24]. Similarly, in the bacterium *C. glutamicum*, enhanced transcription of Zn^2+^ uptake systems has been observed when ethanol is used as a carbon source [1]. In the case of *Zymomonas mobilis*, it has been found that high intracellular concentrations of Zn^2+^ decrease the activity of ADHII, and Fe^+^ is needed for its de novo synthesis [25].

**Fig. 2:**
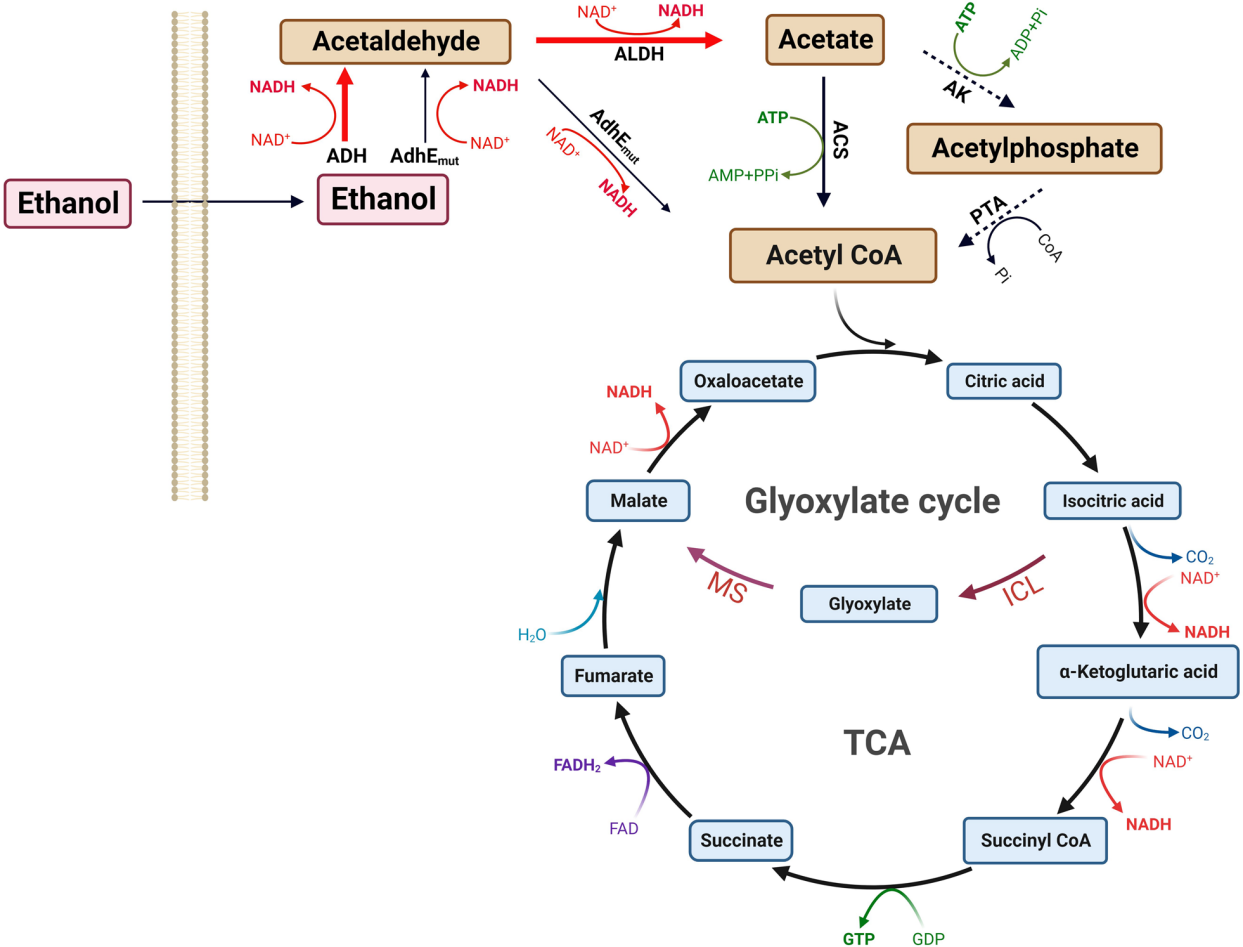
The main ethanol utilization (EUT) pathway in microorganisms. Take NAD^+^-dependent ADH and ALDH as an example, ICL: isocitrate lyase, MS: malate synthase. Figure created with BioRender.com

Acetate is subsequently activated to acetyl-CoA, entering into the TCA and glyoxylate cycles and generating a significant amount of reducing force, energy, and carbon building blocks to support cell growth and metabolism. Microorganisms employ different enzymatic systems to convert acetate into acetyl-CoA during ethanol metabolism. In bacteria, species like *C. glutamicum* leverage a two-step process involving acetate kinase (AK) and phosphotransacetylase (PTA) to convert acetate first into acetylphosphate and then into acetyl-CoA [1, 26]. Alternatively, species like *Pseudomonas putida* use acetyl-CoA synthetases (ACS) for this conversion [27]. Yeast such as *Yarrowia lipolytica* and *Pichia pastoris* also utilize ACS for this conversion [28]. Although the AK–PTK pathway consumes less ATP, the one-step ACS system has been observed to exhibit higher conversion efficiency of acetyl-CoA [14] (Fig. 2). Acetyl-CoA subsequently enters the TCA cycle, linking ethanol metabolism to central metabolic pathways. Here, it can combine with oxaloacetate to form citrate and oxidize to release CO_2_ and generate GTP, NADH, and FADH_2_. When ethanol is the primary carbon source, the glyoxylate cycle becomes more active. The expression of glyoxylate cycle genes, such as isocitrate lyase (ICL) and malate synthase (MS), can be significantly induced by ethanol [26, 29, 30]. Although this bypass generates less energy, it allows for the synthesis of four-carbon compounds like malate and succinate, which are essential for microorganisms growing on ethanol (Fig. 2).

Unique ethanol metabolic pathways have been also identified in certain microorganisms. For example, *E. coli* possesses an endogenous bifunctional alcohol/aldehyde dehydrogenase AdhE for ethanol metabolism. However, *E. coli* cannot metabolize ethanol aerobically due to the low expression of AdhE and its sensitivity to oxygen. A mutated version of AdhE (AdhE ^A267T/E568K^ mutant) enables *E. coli* to overcome this barrier and metabolize ethanol [31]. This unique pathway skips the step from acetaldehyde to acetate, instead directly converting acetaldehyde directly to acetyl-CoA, resulting in a more efficient energy-generating pathway (Fig. 1). Additionally, in *S. cerevisiae*, ethanol degradation can occur through esterification catalyzed by acyl-coenzymeA: ethanol O-acyltransferase, resulting the production of fatty acid ethyl esters (FAEEs) [32]. The discovery of these specific pathways for ethanol metabolism presents new opportunities for future biotechnological applications and offers additional strategies for metabolic engineering.

## Regulation of ethanol metabolism

The primary regulation of ethanol metabolism at the transcriptional level is carbon catabolite repression (CCR), a mechanism widely employed by microorganisms to preferentially utilize certain carbon sources, such as glucose, over less favorable ones like ethanol, lactose, or galactose [1, 33, 34]. In *S. cerevisiae*, a model organism widely used for studying ethanol metabolism, two key transcription factors, Adr1p and Cat8p, play crucial roles in this regulatory process. When glucose is present, the type 1 protein phosphatase (PP1) complex, Glc7p/Reg1p, inhibits the binding of Adr1p to the upstream region of the ADH2 promoter, this gene encodes an ADH that is responsible for converting ethanol to acetaldehyde, thus repressing its transcription [24, 35]. However, once glucose is exhausted, Adr1p and Cat8p bind to specific regions of the ADH2 promoter, with Snf1p positively regulating this Adr1p binding process [24, 36]. Likewise, in the bacterium *C. glutamicum*, the transcriptional regulators of acetate metabolism, RamB and RamA, are crucial for CCR [2, 37]. With glucose present in the medium, RamB binds to the upstream of promoters of multiple genes involved in the ethanol metabolism, including ADH, ALDH, AK, and PTA, inhibiting their transcription. When glucose is removed, RamA acts as a transcriptional activator by binding to these genes and promoting their transcription [37]. Some fungi exhibit oxygen-dependent transcriptional regulation mechanisms. For example, an increase in ADH expression under hypoxic conditions has been observed in *Scheffersomyces stipites* and *Metarhizium acridum* [24]. Chromatin remodeling, a vital process affecting gene accessibility and expression, also contributes to the transcriptional regulation of ethanol catabolism. In *S. cerevisiae*, alterations in nucleosome spacing or histone deacetylation/acetylation can modify the chromosome structure surrounding ADH, thereby influencing ADH mRNA accumulation [35].

While much of current research focuses on the transcriptional level, post-transcriptional and post-translational mechanisms also play crucial roles in the regulation of ethanol metabolism. In *E. coli*, RNase III cleavage is necessary for the translation initiation of AdhE, enabling the ribosome binding site (RBS) to be accessible [38]. Post-translational modifications, including amino acid substitution and Zn^2+^-mediated enzyme activity regulation, have been reported [39, 40]. For instance, in *E. coli*, a substitution from Glu568 to Lys in the AdhE gene can result in the production of active AdhE protein under both aerobic and anaerobic conditions [41]. Zn^2+^ can directly influence the formation of the active center of ADH, thereby impacting the overall metabolic processes [24]. Despite significant advancements in understanding the regulation of ethanol oxidation, particularly at the transcriptional level, further exploration is still needed to fully comprehend this process in microorganisms.

## Ethanol stress

**Fig. 3:**
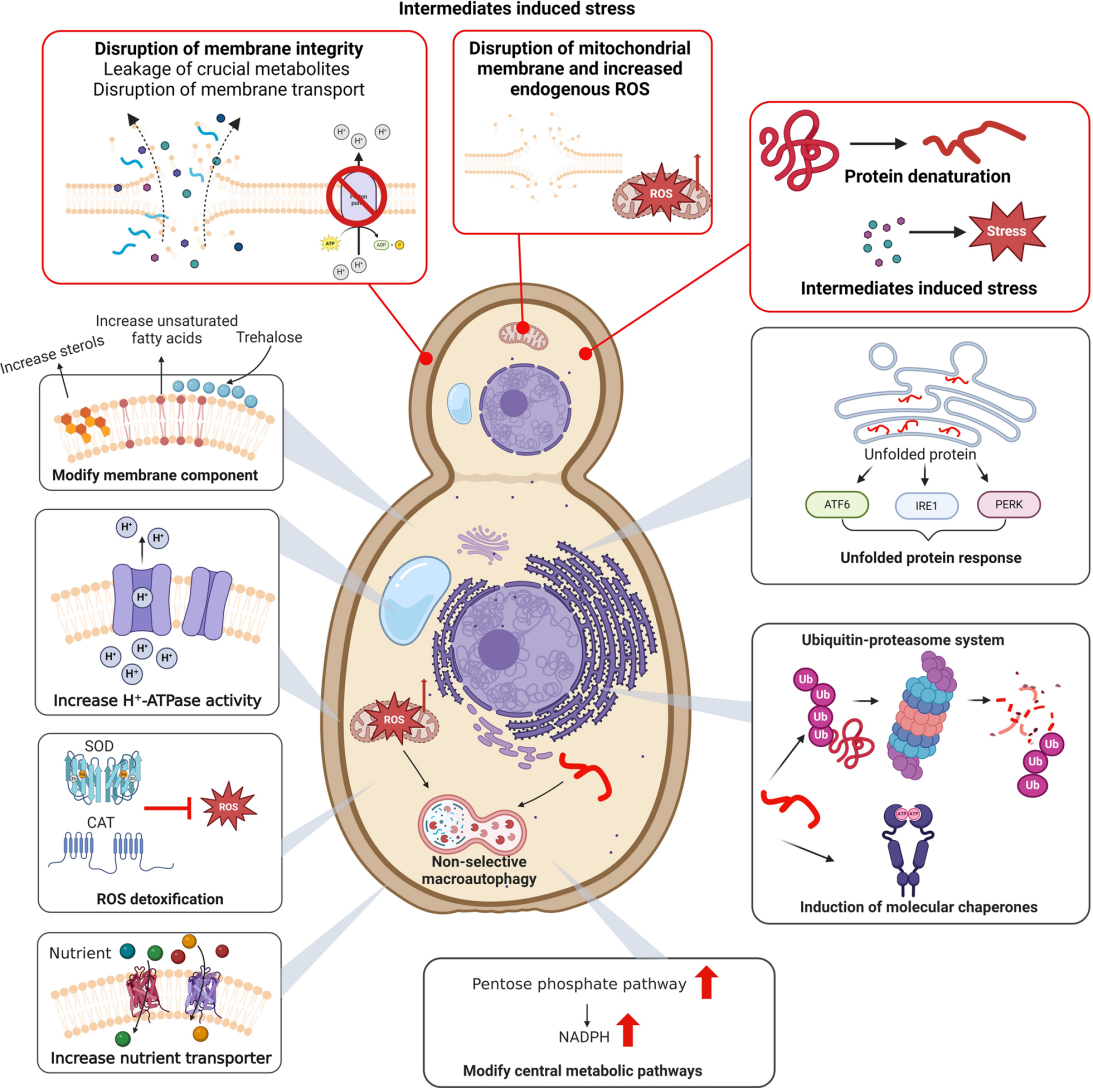
Ethanol stress and stress response in *S. cerevisiae*. The red box represents ethanol stress and the black box represents ethanol stress response. Figure created with BioRender.com

When exposed to high concentrations of ethanol, microbes may experience ethanol stress, which is fundamentally a type of organic solvent stress. This triggers a series of physiological and metabolic changes in microbial cells (Fig. 3).

Directly, ethanol exposure can alter membrane fluidity, permeability, and integrity [42]. Ethanol interacts with lipid components and induces phase transitions, which cause significant damage to membrane structure. This damage results in increased passive proton flux, disrupting the electrochemical gradient essential for nutrient uptake [43–45]. As a result, cells experience a decrease in intracellular pH and plasma membrane depolarization, which impairs nutrient transport [45]. Additionally, the disruption of the membrane can lead to the leakage of critical metabolites, such as amino acids and nucleotides, into the extracellular environment [46].

Ethanol can also disrupt protein structure and cause protein denaturation through various mechanisms. These include disturbing hydrogen bonds among amino acid residues, competing with water for solvation at the protein surface, and interacting with hydrophobic residues [47]. Another consequence of ethanol stress is increased oxidative stress [48]. The mitochondrial electron transport chain produces endogenous reactive oxygen species (ROS) as a byproduct of its function. However, ethanol can impair the tightly regulated process of iron homeostasis within the mitochondria, including the biogenesis and recycling of iron-sulfur clusters [49]. This disturbance can result in the release of free iron and an increase in ROS formation [45]. Ethanol also interacts with mitochondrial membranes, reducing proton motive forces and causing proton leakage [50].

Apart from the stress induced by ethanol itself, its metabolic intermediates—acetaldehyde and acetate—can also potentially induce cellular stress, particularly when the balance of ethanol metabolism is disrupted. Acetaldehyde is highly toxic and reactive, with the potential to damage proteins, DNA, and lipids, thereby impairing growth and metabolism [51]. Although less toxic, acetate can disrupt the transmembrane pH gradient, lead to an accumulation of acetic anions, and interfere with biosynthesis pathways [7]. Therefore, when engineering ethanol metabolic pathways, it is crucial to carefully modulate enzyme activities. This can minimize the accumulation of these intermediate products, which in turn can mitigate the stress response, fostering optimal microbial growth and productivity.

## Ethanol stress response and tolerance

### Ethanol stress response and tolerance in *S. cerevisiae*

*S. cerevisiae*, the most frequently used organism in ethanol production, provides valuable insights into microbial response and tolerance to ethanol stress. When exposed to high ethanol concentrations, *S. cerevisiae* triggers numerous adaptive cellular responses to combat membrane damage, protein denaturation, and increased oxidative stress (Fig. 3).

In response to membrane damage, *S. cerevisiae* increases the content of ergosterol and unsaturated fatty acids in cell membranes [52], accumulates trehalose for osmoprotection [53], and upregulates the activity of H^+^-ATPase for intracellular pH maintenance [54] (Fig. 3). Cells also activate their protein quality control systems such as molecular chaperones, the ubiquitin–proteasome system (UPS), autophagy, and the unfolded protein response (UPR), to overcome ethanol-induced protein misfolding and denaturation [45, 55]. To overcome mitochondrial damage and resist the increased oxidative stress induced by ethanol, *S. cerevisiae* enhances the metabolic flux towards the pentose phosphate pathway (PPP) to produce more redox and energy cofactors. Cells also improve antioxidant enzyme activities, such as superoxide dismutase (SODs) and catalase (CAT), and activate non-selective macroautophagy to combat ethanol-induced ROS [45, 50]. In addition to the above responses, *S. cerevisiae* can alter the concentration of certain amino acids (AA) to further combat ethanol-induced stress and energy deficiency. This involves increasing the concentrations of amino acids such as proline and tryptophan to cope with stressful conditions [46]. To conserve energy, the synthesis of certain amino acids like isoleucine, threonine, aspartate, and glutamate is reduced [43].

Research involving transposon mutants and single-gene knockout experiments has identified hundreds of genes associated with ethanol tolerance in *S. cerevisiae*. These genes cover a broad range of functional categories such as membrane and cell wall composition, amino acid metabolism, trehalose and glycogen metabolisms, cell cycle, protein transport/vacuole, mitochondrial function, and peroxisomal transport [44, 56]. For example, a recent study noted that deletions in LDB19 (endocytosis), MEH1 (vacuolar), PRO2 (proline synthesis), and YNL335W (unknown function) rendered the yeast cells sensitive to ethanol stress. This heightened sensitivity may arise from compromised vacuolar function and amino acid biosynthesis, thereby emphasizing their importance in managing ethanol stress. This study also revealed that the deletion of CYB5 and YOR139C specifically enhances ethanol tolerance. YOR139C is implicated in the repression of flocculation-related genes, while CYB5 participates in sterol and lipid biosynthesis, processes critical for ethanol tolerance [57]. A comprehensive analysis by Stanley et al., which involved a thorough examination of previous independent deletion library screens for *S. cerevisiae* genes associated with ethanol tolerance, revealed that deletions in VPS36 and SMI1, genes associated with vacuole protein sorting and cell wall synthesis, consistently resulted in increased ethanol sensitivity across multiple studies [44].

Recently, the use of multiple omics technologies has further advanced our understanding of the ethanol tolerance mechanism. Unlike previous studies that primarily focused on identifying specific mutations increasing ethanol tolerance, these new methodologies provide a more comprehensive view of the complex regulatory networks involved. Integrated multi-omics data indicate that ethanol-tolerant *S. cerevisiae* strains have reprogrammed numerous metabolic pathways, from energy and lipid metabolism to the regulation of transposons and proteins implicated in cell cycle progression [58]. Under conditions of ethanol stress, ethanol-adapted yeast strains show a significant shift in energy metabolism, relying more heavily on glycolysis and ethanol fermentation for energy production. In contrast, the ancestral strain transitions from ethanol fermentation to respiration. These changes emphasize the critical role of metabolic adaptation in the mechanism of ethanol tolerance [58]. Proteins differentially regulated in these ethanol-adapted yeast strains and their associated cellular functions are summarized in Table 2.

**Table 2:** Specifically regulated proteins in ethanol-tolerant yeast strains and the related cellular processes

Cellular processes	Protein^a^
Ribosome biogenesis	RIO2, RIO1, **TSR2**, **YAR1**, SFM1, HMT1, RMT2, EFM3, **RSA3**, YVH1
RNAPol II preinitiation complex assembly	SPT15, **NCB2**
Ion homeostasis	CMP2, CNA1
Cell wall integrity	TOR2, PKC1, PPZ1, PPZ2
Cell cycle	CDC14, PKC1
Heat response	HSF1, TOR1
Glucose genes regulation	GLC7, SNF1
mRNA polyA tails	SSU72, **FIP1**, GLC7
Telomere maintenance	RAP1, TBF1
Chromatin remodeling	RSC30, RSC3, **RTT102**
DNA damage checkpoint and repair	MEC1, TEL1
AA synthesis	GCN4, **LEU2**
Autophagy	**ATG8**, ATG1,VPS15, TPK1, VPS34
Fatty acids	FAA4, FAS1
TCA and glyoxylate cycle	RTG3, **CIT2**, PTC6, RTG1
Ubiquitin proteasome pathway	DSK2, HRD1, SSM4, UBC4, TOM1, UBC8
L-Met salvage pathway	ADI1, ARO9, ARO80
Oxidative stress	MXR1, CAM1, TMA19
Purine synthesis	APT1, HPT1
Protein import to mitochondria	SAM50, TIM50
Import of extracellular AAs	AGP1, STP1
Other	**SCP1**, PTP1, PPQ1, GAL4, FHL1, UBA4, PIB1, MOT3,

^a^ Red character indicates coding genes containing single nucleotide polymorphisms (SNPs), bold character indicates regulatory protein

### Ethanol stress response and tolerance in *bacteria*

The ethanol stress response and tolerance mechanism in bacteria has also been studied. To combat the detrimental effects of higher ethanol concentrations, *E. coli* employs a series of defensive strategies. These include upregulating ABC transporters, altering membrane composition, increasing heat-shock protein production, and adjusting central metabolism (e.g., boosting propionic acid metabolism and the TCA cycle and minimizing non-essential ATP consumption) [59, 60]. The global regulator, cAMP receptor protein (CRP), also plays a crucial role in this process by activating essential biosynthetic pathways for ethanol tolerance [61, 62].

Further insights can also be gained from studies on other bacteria. For instance, *Z. mobilis*, when subjected to ethanol stress, increases the hopanoid content in its membranes to strengthen the hydrophobic barrier. Additionally, it reduces the phospholipid ratio, which decreases the surface area of lipid patches available for passive leakage, thus improving ethanol tolerance [60, 63]. Gram-positive bacteria like *Lactobacillus heterohiochii*, known for their ethanol tolerance, possess long-chain monounsaturated phospholipids (C20–30), which contribute to their ability to withstand high ethanol concentrations [60]. In *C. glutamicum*, a single nucleotide variation (SNV) identified upstream of the ald gene has been found to enhance ethanol utilization, potentially contributing to improved ethanol tolerance [64].

Despite significant progress, current research primarily focuses on identifying genes associated with microbial ethanol stress and tolerance. It is now crucial to delve deeper to ascertain precisely how these associated genes influence ethanol tolerance and to comprehend the underlying mechanisms that govern this tolerance. Achieving this advanced understanding may be possible through a systems-level approach that integrates adaptive laboratory evolution, multi-omics analysis, and advanced computational techniques, such as machine learning and artificial intelligence (AI) [65, 66]. This integrated approach has the potential to dissect the complexity of ethanol tolerance at multiple biological levels—from genes to pathways to systems. By deepening our understanding of these mechanisms, we can develop more robust and efficient microbial strains for ethanol-based biomanufacturing.

## Ethanol-based biomanufacturing

### Microbial chassis

Ethanol-based biomanufacturing is a process that leverages the metabolic ability of certain microbes to efficiently convert ethanol into valuable bioproducts [14, 21, 67]. The ideal microbial chassis for this process should exhibit three key features: (1) the metabolic capability to use ethanol as a carbon and energy source, (2) a certain degree of ethanol tolerance, and (3) amenability to genetic modification.

**Fig. 4:**
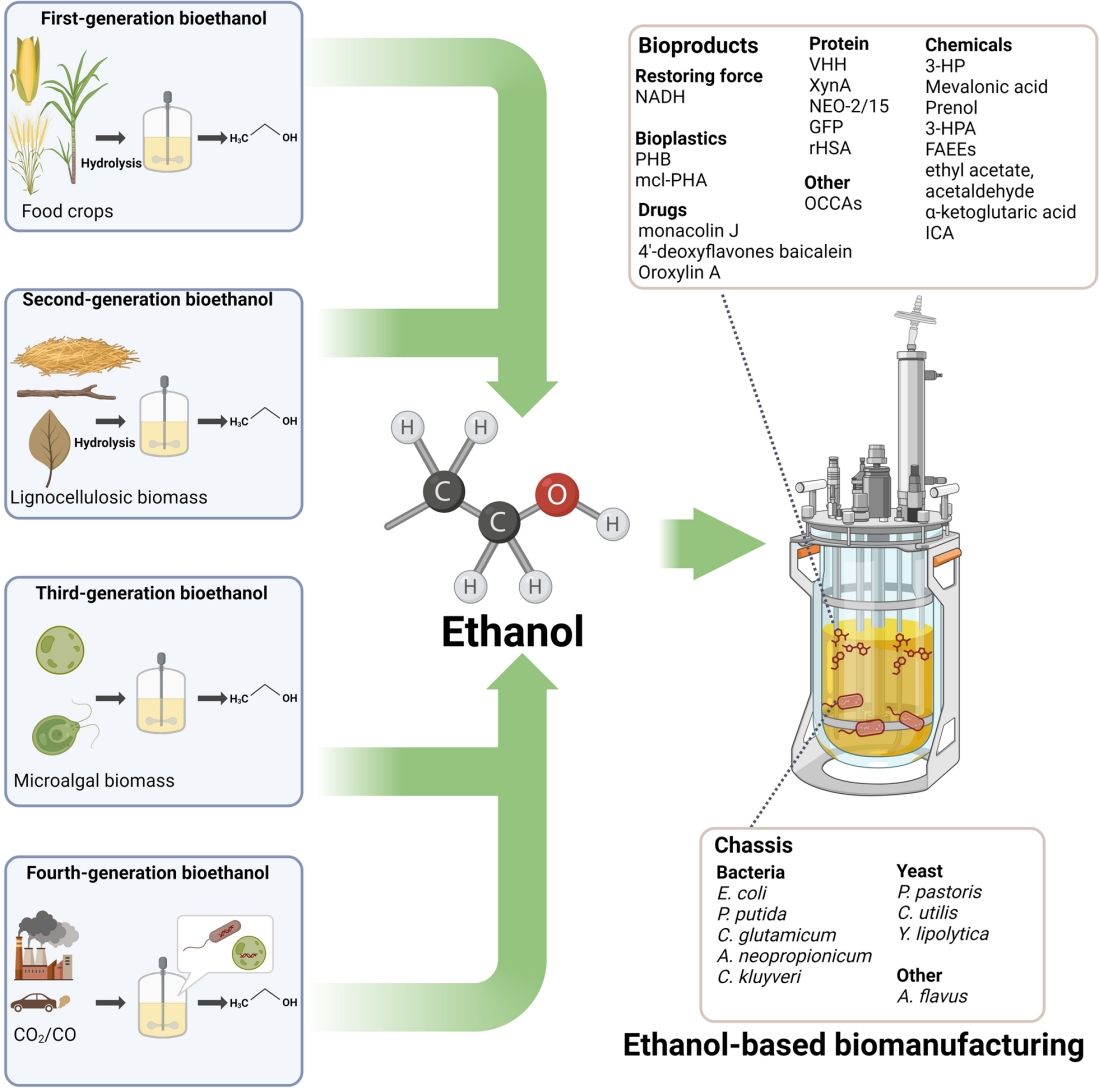
Bioethanol synthesis and ethanol-based biomanufacturing**.** Figure created with BioRender.com

Microbial strains such as* P. putida*, *C. glutamicum*, *P. pastoris*, and *Y. lipolytica* naturally possess the ability to metabolize ethanol and have been employed as cell factories in ethanol-based biomanufacturing (Table 3, Fig. 4). In the case of *P. putida,* advancements have been made in boosting its efficiency in assimilating ethanol. This has been achieved by substituting the native two-step convention from acetaldehyde to acetyl-CoA with a single-step reaction facilitated by acetaldehyde dehydrogenases acylating (Ada) [27, 67, 68]. Moreover, the heterogeneous expression of ACS and eutE (putative aldehyde dehydrogenase) has been explored to enhance the process of converting ethanol into acetyl-CoA in *P. putida* [69]. The spectrum of potential chassis cells has been further expanded through genetic engineering, allowing organisms that naturally do not metabolize ethanol under certain conditions, like *E. coli* in aerobic conditions, to gain this ability. These engineering efforts typically involve modifying the native pathway or introducing heterologous pathways [70, 71]. However, it is worth noting that an enhanced ethanol metabolism can often lead to high oxygen demand. To mitigate this challenge, strategies such as expressing vitreous hemoglobin (VHb) can be adopted to help boost ethanol metabolism under low oxygen conditions, thereby reducing the need for oxygen supply [72].

**Table 3:** Ethanol-based biomanufacturing

Organism^a^	Strategy	Medium	Product	Yield (g/L)	Reference
Bacteria					
*E. coli*	Introducing exogenous ADH gene from *Gluconobacter thailandicus*	Co-culture, 2% ethanol	Produce NADH for xylitol production	\	[70]
*E. coli*	Expressing native AdhE ^A267T/E568K^ mutant	Minimal medium + 10 g/L glucose + 10 g/L ethanol	Poly(3-hydroxybutyrate) (PHB), 3-hydroxypropionic acid (3-HPA), phloroglucinol (PG)	35.67 g/L PHB (bioreactor level), 0.50 ± 0.01 g/L 3HP (flask), 0.38 ± 0.02 g/L PG (flask)	[14]
*E. coli*	Introducing the ADH and ALDH from *Aspergillus nidulans*	M9 + 5 g/L ethanol	Mevalonic acid	\	[71]
*E. coli*	Introducing exogenous ADH gene from *S. cerevisiae* and acetaldehyde dehydrogenase (acylating) (Ada) from *Dickeya zeae*	K3 medium + 10 g/L ethanol	PHB, prenol	1.1 g/L of PHB (flask) 24 mg/L of prenol (flask)	[67]
*E. coli*	Expressing native AdhE ^A267T/E568K^ mutant, or co-expressing AdhP and acetylating aldehyde dehydrogenase mutant MhpF_Mut_	M9 minimal salt medium containing 10 g/L ethanol	3-HPA	1.66 g/L (flask), 13.17 g/L (whole-cell biocatalysis system)	[76]
*Pseudomonas putida*	Native pathway accompanied by a heterogeneous expression of acs and eutE	Modified M9 media with 10 g/L of ethanol and 1 g/L of dextrose	Mevalonic acid	0.41 g /g ethanol (flask), 0.32 g /g ethanol (bioreactor)	[69]
*Pseudomonas putida*	Native pathway accompanied by a heterogeneous expression of Ada	M9 medium containing 1% ethanol	Fatty acid ethyl esters (FAEEs)	1.6 g/L (shake flask), 4.3 g/L (bioreactor)	[27]
*Pseudomonas putida*	Native pathway accompanied by a heterogeneous expression of Ada and pduP	M9 medium containing 1% ethanol	PHB	1385.34 ± 16.5 mg/L (flask), 4.3 g/L (bioreactor)	[68]
*Pseudomonas putida*	Native pathway	Digestate medium with about 2 g·L^–1^·h^−1^ ethanol feeding	Medium chain length polyhydroxyalkanoate (mcl-PHA)	6 g/L (bioreactor)	[75]
*Corynebacterium glutamicum*	Native pathway	LBB + 1% ethanol	VHH, XynA, NEO-2/15	281 mg/L (NEO-2/15, flask)	[26]
*Anaerotignum neopropionicum and Clostridium kluyveri*	Native pathway	Co-culture, 50 or 90 mM ethanol	Valerate and heptanoate	\	[80]
Yeast					
*P. pastoris*	Native pathway	YNB + 0.5% ethanol	Monacolin J	2.2 g/L (bioreactor)	[19]
*P. pastoris*	Native pathway	ASMv6 medium with different ethanol concentrations	Recombinant human serum albumin (rHSA)	1.13 mg/g_WCW_^**b**^	[79]
*P. pastoris*	Native pathway	YPE medium containing 0.5% ethanol	Baicalein, Oroxylin A	401.9 mg/L (baicalein, flask), 339.5 mg/L (Oroxylin A, flask)	[21]
*Candida utilis*	Native pathway	Iron-free salt medium containing 16 g/L or 32 g/L ethanol	Ethyl acetate	\	[82]
*Yarrowia lipolytica*	Native pathway	Medium containing 2–6 g/L ethanol	Isocitric acid (ICA)	90.5 g/L (bioreactor)	[78]
*Yarrowia lipolytica*	Native pathway	Basal mineral medium containing less than 2.5 g/L ethanol	α-Ketoglutaric acid	49.0 g/L (bioreactor)	[77]
Other strains					
*Aspergillus flavus*	Native pathway	Potato dextrose broth liquid medium with low-dose ethanol (< 1%)	Aflatoxin	\	[81]

^a^ Ethanol tolerance in different chassis. *E. coli*, grow very little in ethanol concentrations above 6% (V/V), preferably below 1% [60]. *P. putida*, can tolerate 5% (V/V) ethanol, preferably 1% [27]. *C. glutamicum*, a final OD_600_ of 5 can reach on 4% (V/V) ethanol, preferably below 2.5% [1, 26]. *A. neopropionicum*, growth decreases sharply when ethanol concentration exceeds 300 mM, preferably 50 mM [80]. *C. kluyveri*, growth can still be sustained well even at a high ethanol concentration of 700 mM [80]. *P. pastoris*, growth is approximately reduced by 50% under 5% (V/V) ethanol conditions, preferably 1%-2% [79]. *C. utilis*, 32 g/L ethanol [82]. *Y. lipolytica*, 2% (V/V) ethanol [89]. *A. flavus*, below 1% (V/V) ethanol [81]. ^b^g_WCW_ represents gram of wet cell weight

Ethanol tolerance is another critical attribute of chassis for ethanol-based biomanufacturing. Organisms such as *P. putida* and *C. glutamicum* have demonstrated moderate ethanol tolerance, exhibiting growth in minimal medium containing 5% and 4% (v/v) ethanol, respectively [1, 27]. Enhancing the ethanol tolerance of these and potential chassis cells involves a variety of strategies. One such strategy is genetics and membrane engineering, which may involve adjustments to the fatty acid chain length and the ratio of unsaturated fatty acids [73]. Other effective options include techniques such as adaptive laboratory evolution (ALE), overexpression of protective metabolites and molecular chaperones, and global transcription machinery engineering (gTME) [73, 74]. Furthermore, improved ethanol utilization can indirectly enhance ethanol tolerance. By metabolizing ethanol more efficiently, cells can reduce both intracellular and extracellular ethanol concentrations, promoting cell survival and functionality in environments with high ethanol concentrations.

### Ethanol-based bioproducts

Table 3 provides a comprehensive overview of recent innovations in the production of ethanol-derived bioproducts. Generally, these bioproducts can be classified into the following categories: (1) acetyl-CoA derivatives, (2) direct intermediates of glyoxylic acid and the TCA cycle, (3) products synthesized through introduced metabolic pathways, and (4) recombinant proteins.

The use of ethanol as a carbon source offers unique advantages, especially in the production of acetyl-CoA. Unlike glucose, ethanol does not lead to carbon loss during catabolism and generates more NADH compared to acetate (Fig. 1). These benefits have drawn significant attention to the production of acetyl-CoA derivatives in ethanol-based biomanufacturing. Bioplastics such as poly (3-hydroxybutyrate) (PHB) and medium-chain length polyhydroxyalkanoate (mcl-PHA), which are derived from acetyl-CoA, have been produced from ethanol in several studies [14, 67, 68, 75]. For instance, Sun et al. engineered the AdhE gene in *E. coli*, thus creating an optimized production strain that achieved a PHB yield of 35.67 g/L under fed-batch fermentation [14]. In a similar effort, Liu et al. aimed to produce Monacolin J, an acetyl-CoA-derived drug and a precursor to the cholesterol-lowering medication simvastatin, in *P. pastoris*. They employed an ethanol-induced transcriptional signal amplification device (ESAD) to enhance monacolin J production, ultimately achieving a titer of 2.2 g/L on ethanol in a bioreactor [19]. Other acetyl-CoA derivatives, such as 3-hydroxypropionic acid (3-HPA) and phloroglucinol (PG), have also been synthesized in *E. coli* [14, 76]. Furthermore, Lu et al. enhanced the 3-HPA yield in *E. coli* using a whole-cell biocatalysis system, reaching a production level of 13.17 g/L [76].

Direct intermediates of glyoxylic acid and the TCA cycle, such as isocitric acid (ICA) and α-ketoglutaric acid, have been successfully synthesized using ethanol as a carbon source in *Y. lipolytica*, achieving yields of 90.5 g/L and 49.0 g/L in a bioreactor, respectively [77, 78]. The introduction of wholly or partially heterologous biosynthetic pathways into microbial chassis has also facilitated the synthesis of high-value chemicals like mevalonic acid, baicalein, and oroxylin A [21, 69]. Ethanol has also been used as a feedstock to facilitate high-level production of recombinant proteins. These proteins include the variable heavy chain of heavy-chain antibodies (VHH), endoxylanase (XynA), the “de novo design” protein NEO-2/15, and recombinant human serum albumin (rHSA) [26, 79]. In addition, other products, such as xylitol, fatty acid ethyl esters (FAEEs), valerate, heptanoate, ethyl acetate, and aflatoxin have been reported to be synthesized using ethanol as a carbon source [27, 70, 80–82].

## Ethanol biosynthesis

Ethanol is a versatile compound with significant applications across various industries such as healthcare, energy, and the chemical sector. With the continual advancements in ethanol-based biomanufacturing, the potential for ethanol to serve as a primary substrate is greatly amplified. The prospective large-scale demand from industrial bioprocessing, coupled with the already substantial market, underscores the criticality of developing efficient, sustainable, and scalable techniques for ethanol production.

Ethanol production methods are primarily divided into chemical synthesis and biosynthesis. Chemical synthesis, often involving the hydration of ethylene, relies heavily on non-renewable resources like oil and natural gas. While biosynthesis typically employs the fermentation of sugars by microorganisms, the production of bioethanol has evolved through several "generations" [83]. The first generation of bioethanol production utilized food crops like corn and sugarcane as feedstocks. However, the use of these food crops raised concerns about competition with food production and the occupation of arable land.

In the face of the challenges of sustainable development and environmental protection, second-generation bioethanol production has been developed [8]. This approach utilizes carbohydrates from non-food biomass—particularly lignocellulosic materials such as agricultural residues and energy crops [83, 84]. This method offers significant advantages over first-generation bioethanol by reducing competition with food crops for land and resources, mitigating greenhouse gas emissions, promoting a circular economy through waste reduction and resource maximization, and providing additional income opportunities for farmers. Through years of research, pretreatment methods have been developed to effectively separate carbohydrates from lignin, which has greatly improved the hydrolysis efficiency of cellulose and hemicellulose into fermentable sugars [84, 85]. Furthermore, several strains of *Saccharomyces cerevisiae*, *E. coli*, and *Z. mobilis* have been developed to efficiently convert these sugars into ethanol [86, 87].

The continuous quest for more sustainable and efficient methods led to the emergence of third-generation bioethanol production. This generation leverages microalgae and cyanobacteria biomass for ethanol production. Algae do not require arable land or fresh water for cultivation. They can grow in wastewater or water with a high salinity content, providing a more environmentally friendly approach without competition with agricultural activity [83, 88]. Algal bioethanol production also has the potential advantage of contributing to carbon dioxide sequestration, as algae absorb CO_2_ during photosynthesis. Over the past decades, numerous studies have optimized growth conditions for maximizing carbohydrate yield and explored efficient harvesting and extraction methods [88]. Integrations of microalgal biomass cultivation with wastewater treatment or industrial waste gas have also been explored to reduce production costs [88].

Recently, the development of genetic engineering and synthetic biology techniques has catalyzed the rise of fourth-generation bioethanol. This new wave of bioethanol leverages waste gases like CO and CO_2_ as primary carbon feedstock. Genetically modified organisms, such as acetogens and cyanobacteria, serve as biocatalysts to convert these waste gases directly into ethanol [8, 15, 90]. This approach avoids the process of cultivating, harvesting, and pretreatment of biomass resources in the third-generation process, effectively reducing energy consumption, equipment requirements, and carbon emissions in the production process, offering a more sustainable solution.

## Conclusions and prospects

The shift towards renewable and non-food feedstocks for bioprocessing has become increasingly urgent due to the global challenges of escalating population growth and food scarcity. Ethanol, as a main product of CO_2_ fixation in third-generation (3G) biorefineries [91], exhibits advantages and uniqueness as a carbon source, including higher mass transfer, easy assimilation by industrial workhorse microorganisms, can be fed into bioreactor in pure form, and great potential in producing acetyl-CoA derivatives [14, 91]. Over the past decade, significant progress has been made in ethanol-based biomanufacturing. These advancements have facilitated the production of a wide array of products, ranging from plastics and chemicals to pharmaceuticals and recombinant proteins, in various organisms using ethanol as the carbon source (Table 3).

Despites these achievements, numerous challenges remain before efficient ethanol-based biomanufacturing can be realized. One major challenge is the development of cost-effective and efficient technologies for bioethanol production from microalgal biomass and waste gases (e.g., CO, CO_2_, and CH_4_) [92]. Although substantial advancements have been made in third-generation and fourth-generation ethanol technologies, the production of ethanol still predominantly relies on sugar-based feedstocks. To address this challenge, genetic engineering and metabolic engineering could be applied to increase the carbohydrate content of microalgae or their ability to directly synthesize ethanol. Additionally, the development of more efficient algal cultivation systems that maximize the exposure of microalgae to sunlight and CO_2_ is necessary. For fourth-generation bioethanol, advancements in gas fermentation technologies are needed to ensure efficient gas-to-liquid transfer. It is advisable to integrate the gas fermentation system with existing infrastructure, such as steel mills or power plants that emit rich CO and CO_2_ waste gases.

Another potential challenge involves integrating ethanol utilization pathways into heterologous strains, which may lead to metabolic imbalances and the accumulation of toxic intermediates like acetaldehyde or acetate. The development of precise and reliable gene expression tools is critical to mitigate these issues by enabling fine-tuned control of gene expression within these pathways, thereby reducing toxic intermediates buildup [9, 93]. Integrating feedback regulation systems directly into metabolic pathways could help maintain balance and prevent the excessive accumulation of toxic metabolites [94]. The compartmentalization of ethanol metabolic pathways within specific cell organelles could also prevent the entry of toxic metabolites into the cytoplasm [95]. Achieving efficient ethanol-based growth also presents challenges, as organisms that naturally use ethanol as their sole carbon source often exhibit slower growth rates compared to those using sugar-based carbon sources. ALE combined with inverse metabolic engineering could identify beneficial mutations that enhance ethanol metabolism and tolerance [96]. In large-scale cultivation, employing a fed-batch strategy could alleviate the growth inhibition effects of high ethanol concentrations, leading to more robust biological transformation processes.

In addition to the above challenges, the reliable scale-up of ethanol-based biomanufacturing is not an easy task. Given the significant heat release and increased oxygen demand from ethanol metabolism, the bioreactor should be carefully designed to ensure adequate oxygen supply and efficient heat management. Considering ethanol's flammable nature, especially in large volumes, robust safety protocols and control systems are also essential.

To fully leverage ethanol's potential, its unique traits, such as CCR-regulated metabolism, heat generation during use, and the limited number of natural utilizers, can be harnessed for various benefits. These include decoupling glucose-based growth from ethanol-driven production phases [19, 26], leveraging heat for electricity co-generation, and developing ethanol-based antibiotic-free systems, etc. [97]. Additionally, exploring underexplored bacteria and microbial species as potential chassis, and creating synthetic microbial communities are necessary for unlocking new opportunities in ethanol-based biomanufacturing [98]. Ethanol stress might exhibit a hormesis effect, which can also be used to enhance product titer across different microbial chassis by introducing a certain concentration of ethanol [99]. To reduce energy and operational costs and enhance the overall sustainability, sequential fermentation can be implemented in the same bioreactor to transform the ethanol into a variety of higher-value products, following the removal of ethanol-producing microbes from the fermentation broth [100–102]. Co-cultivation with ethanol-producing algae is another viable strategy that merits consideration [100–102]. Finally, ensuring that ethanol-based biomanufacturing is sustainable and aligns with global sustainability goals is vital. A comprehensive environmental impact assessment, including factors like water and energy consumption, emissions, and potential air pollutants, should be conducted. Utilizing a life cycle analysis (LCA) would help evaluate the overall sustainability and establish ethanol as a viable alternative to sugar-based carbon sources [103].

## Data Availability

No datasets were generated or analysed during the current study.
